# The Impact of Nutrition and Environmental Epigenetics on Human Health and Disease

**DOI:** 10.3390/ijms19113425

**Published:** 2018-11-01

**Authors:** Céline Tiffon

**Affiliations:** French National Cancer Institute, 92100 Boulogne-Billancourt, France; celine.tiffon@gmail.com; Tel. +33-(0)1-4110-1614

**Keywords:** epigenetic marks, signal integration, molecular mechanisms, nutrients, endocrine disruptors, mammary gland, personalized health

## Abstract

Environmental epigenetics describes how environmental factors affect cellular epigenetics and, hence, human health. Epigenetic marks alter the spatial conformation of chromatin to regulate gene expression. Environmental factors with epigenetic effects include behaviors, nutrition, and chemicals and industrial pollutants. Epigenetic mechanisms are also implicated during development in utero and at the cellular level, so environmental exposures may harm the fetus by impairing the epigenome of the developing organism to modify disease risk later in life. By contrast, bioactive food components may trigger protective epigenetic modifications throughout life, with early life nutrition being particularly important. Beyond their genetics, the overall health status of an individual may be regarded as an integration of many environmental signals starting at gestation and acting through epigenetic modifications. This review explores how the environment affects the epigenome in health and disease, with a particular focus on cancer. Understanding the molecular effects of behavior, nutrients, and pollutants might be relevant for developing preventative strategies and personalized heath programs. Furthermore, by restoring cellular differentiation, epigenetic drugs could represent a potential strategy for the treatment of many diseases including cancer.

## 1. Introduction

### 1.1. A Brief Introduction to Epigenetics

Epigenetics refers to the control of gene expression via mechanisms not directly related to the DNA coding sequence [1]. As a result, all cells in an organism have very different phenotypes despite having the same genome. Epigenetics modulates and regulates gene expression through various epigenomic “marks”, the term given to chemical compounds added to DNA or histone proteins and recognized by enzymes that either lay down or remove the specific mark. These marks change the spatial conformation of chromatin: either compacting it, thereby preventing the binding of transcription factors to the DNA, or opening it, allowing transcription factor binding and usually upregulating cellular processes.

DNA methylation—the addition of methyl groups to the 5-carbons of cytosine residues in CpG islands to give rise to 5-methylcytosines—works together with histone modifications to regulate gene expression. DNA methylation tends to act at promoters to induce gene silencing, while histone acetylation usually unwinds chromatin. DNA methylation is catalyzed by enzymes in the DNA methyltransferase (DNMT) family, which recruit functional complexes containing DNA methylation domains, leading to transcriptional inhibition or maintaining a repressive chromatin state.

Histone acetylation is associated with transcriptional activity and an open chromatin state [2,3]. Acetylation of histone tails is controlled by two enzyme families: histone acetyltransferases (HATs), which transfer an acetyl group, and histone deacetylases (HDACs) [4,5,6], which remove acetyl groups. Other epigenetic marks are also described and include histone post-translational modifications such as methylation, ubiquitination, sumoylation, phosphorylation, biotinylation, and ADP-ribosylation, which either promote or suppress gene expression. The pattern of these marks on histone tails is often referred to as the histone code, which dictates the binding of effector proteins that in turn results in specific cellular processes.

Non-coding RNAs (ncRNAs) are another type of specific epigenetic mark that mediate various intracellular processes [7]. A ncRNA is a functional RNA molecule transcribed from DNA but not translated into protein. The best characterized ncRNAs are microRNAs (miRNAs), which are short, single-stranded, 19–24 nucleotide ncRNAs. miRNAs regulate gene silencing at the transcriptional and/or translational level of protein-coding genes [8]. Long non-coding RNAs (lncRNAs) are another subset of RNAs over 200 nucleotides in length that function as chromatin remodelers, transcriptional regulators, and post-transcriptional regulators. Many lncRNAs complex with chromatin-modifying proteins to recruit their catalytic activity to specific genomic sites, thereby modifying chromatin states and influencing gene expression. With little or no protein-coding potential, lncRNAs instead participate in various intracellular processes [7], and recent studies have identified that certain lncRNAs are specifically associated with certain cancers [9].

With the development of high-resolution sequencing and high-throughput technologies, a large number of biologically functional ncRNAs have now been identified [10]. In addition, there is crosstalk between DNA methylation and histone modifications [11]; for example, histone methyltransferases, histone demethylases, and accessory proteins interact and coordinate the chromatin state and DNA methylation and methylation status of histones are tightly associated [12]. Moreover, various ncRNAs are also closely associated with other epigenetic marks, which form extensive crosstalk throughout the cell, or the “epigenetic network” [7]. There is now plenty of evidence that the epigenetic control of the genome is far more complicated than first thought and involves multiple epigenetic mechanisms and their interactions [12,13].

More recently, histone variants, mostly of canonical histones H2A, H2B, and H3, with specific properties have been identified in humans and other higher eukaryotes. Most of them are H2A variants, among which macroH2A1 generates alternative splice isoforms, i.e., macroH2A1.1 and macroH2A1.2. MacroH2A1 isoforms appear to be critical regulators of chromatin structure and chromatin dynamics during cellular senescence, regeneration, and fasting [14,15]. The function of these histone variant proteins and their molecular mechanisms in health and during an organism’s lifespan are reviewed in [14]. The discovery of histone variants and their diversity has added further complexity to context-dependent biological systems and their regulation, such as during health, aging, and in pathological conditions including cancer. This is an area of ongoing research, since chromatin dynamics throughout life are likely to change and the epigenome suffers from a progressive loss in configuration during aging [15]. The resulting abnormal chromatin state during aging is characterized by different incorporated histone variants, nucleosome remodeling, altered histone modification patterns, and altered DNA methylation patterns, resulting in the recruitment of different chromatin modifiers, abnormal gene expression patterns, and genomic instability. Among the multiple different variants of histone H2A, macroH2A has been implicated in aging; its level increases in an age-dependent manner during replicative senescence in cultured human fibroblast cells and also in several tissues of aged mice and primates [15].

### 1.2. Environmental Epigenetics

These active or repressive marks are also dependent on lifestyle and environmental factors. “Environmental epigenetics” refers to how environmental exposures affect epigenetic changes [16]. Life experiences, habits, and our environment shape what and who we are by virtue of their impact on our epigenome and health; for instance, although identical twins share the same genome and are superficially phenotypically similar, they are unique individuals with definable differences. These differences result from distinct gene expression influenced by epigenetic factors. Behavior, nutrition, and exposure to toxins and pollutants are among the lifestyle factors known to be associated with epigenetic modifications. For example, nutrition is a key environmental exposure from gestation to death that impacts our health by influencing epigenetic phenomena. In another example, recent epidemiological data suggest that the increased incidence of cancer observed in the developed world since the 1960s may partly be due to exposure to endocrine-disrupting chemicals (EDCs), to which humans and wildlife are exposed daily from multiple sources. These are discussed in detail below.

### 1.3. Cancer: Current Status and Future Prospects

Cancer is the second most common cause of death in most countries and will remain so as elderly people are most susceptible to cancer and the population is rapidly aging, at least in the west. While age-standardized cancer mortality rates are projected to decrease in the European Union (EU) [17,18] and the United States (US) [19,20] due to advances in screening, prevention, and treatment, the incidence has increased in Europe and the US for testicular and prostate cancers over the last 50 years [21,22,23]. In the United Kingdom (UK), the combined incidence of all cancers has increased for all age groups since the early 1990s, with the greatest increase seen in young people aged 0–24 years.

Cancer is a genetic disease characterized by inherited or sporadic mutations in genes that maintain tissue homeostasis, control the cell cycle, or regulate apoptosis. Cancer is also an epigenetic disease characterized by mutations in chromatin-remodeling enzymes and epigenome alterations resulting from aberrant attachment or removal of DNA or histone protein marks. Accumulating evidence suggests that many adult diseases, including cancers, have epigenetic origins.

### 1.4. Article Overview

This review explores the effects and consequences of lifestyle and environmental exposures from gestation and beyond on human health via epigenetic modifications. How epigenetic targets are affected by lifestyle and environmental factors, especially nutritional factors and chemical exposure, are discussed in detail. Understanding the underlying molecular mechanisms and deciphering the signaling pathways involved in environmental epigenetics and disease risk paves the way for personalized interventions such as personalized nutrition or risk management plans, with cancer used as the main illustrative model.

## 2. Nutritional Epigenetics

### 2.1. Nutritional Epigenetics in Health and Disease

Nutrition is one of the most studied and better understood environmental epigenetic factors. Associations have been observed between adverse prenatal nutritional conditions, postnatal health, and increased risk of disease. For instance, at its extreme, the Dutch Famine Birth Cohort resulting from the Dutch Famine of 1944–1945 has been used to study the effects of starvation during pregnancy and subsequent health and developmental outcomes including, but not limited to, increased risk of type II diabetes mellitus, cardiovascular disease, metabolic disorders, and decreased cognitive function in later life [24,25,26]. The first months of pregnancy seem to have the greatest effect on disease risk; children conceived during the Dutch Famine tended to have smaller-than-usual offspring, suggesting that effects may persist and impact our children and even beyond. It seems likely that the fetus epigenetically adapts in response to a limited supply of nutrients. In humans, persistent epigenetic differences associated with prenatal exposure to famine have been ascribed to a lower degree of methylation of a gene implicated in insulin metabolism than their unexposed siblings [27].

The evidence for transgenerational effects of poor maternal diet on human populations with respect to metabolic outcomes was examined in [28]. There is evidence from historical records that the grandchildren of women exposed to famine and other dietary alterations during pregnancy are more likely to experience health complications than their control counterparts. The potential molecular mechanisms of transgenerational inheritance suggest methylation of gametes via the paternal and maternal lineage [28]. Indeed, further transmission via the paternal line is highly likely to occur via epigenetic modulation of the spermatozoan nucleus. Two examples from historical cohorts illustrate this transgenerational transmission via the paternal lineage. One showed that female grandchildren (F2) from the paternal grandmother (F0) who experienced poor food availability during her own growth were at higher risk of cardiovascular mortality. The second more recent example showed that adult grand-offspring whose fathers were exposed to famine in utero had higher BMIs than a control population. The evidence that both maternal and paternal diets influence metabolic phenotypes in offspring in mammals through epigenetic information transmission is reviewed in [29]. Over molecular mechanisms with respect to the fetal origins of adult disease have been suggested including mitochondrial dysfunction and oxidative stress as among the earliest events described in offspring exposed to nutrient restriction [29].

Nutrition in early life induces long-term changes in DNA methylation that impact on individual health and age-related diseases throughout life [11,30]. Nutrients can either act directly by inhibiting epigenetic enzymes such as DNMT, HDAC, or HAT or by altering the availability of substrate necessary for those enzymatic reactions. This in turn modifies the expression of critical genes and impacting on our overall health and longevity (see [12,31] for reviews).

A number of studies have reported the epigenetic effects of diet on phenotype and susceptibility to diseases throughout life. Folate metabolism is linked to phenotypic changes through DNA methylation, as folate, a water-soluble B vitamin, is a source of one-carbon for the synthesis of AdoMet, which is necessary for DNA methylation [12,30,31]. Other methyl donor nutrients such as choline can also alter the DNA methylation status and subsequently impact gene expression [12]. Maternal methyl donor nutrient availability in early pregnancy is essential for proper fetal development, with consequences for health and disease susceptibility or cancer in the children throughout life. In one animal study, a maternal diet restricted in methyl nutrients during periconception affected DNA methylation patterns in offspring and was the cause of altered phenotypes [12].

Conversely, dietary restriction but without severe nutritional deprivation has been shown in several models to extend lifespan [32]. Calorie restriction has an anti-inflammatory effect through the inhibitory effects of critical genes not limited to NF-κB [12]. From the epigenetic standpoint, there is clear crosstalk between DNA methylation and histone modifications [11], suggesting that the chromatin structure may also determine DNA methylation [11]. In this context of epigenetic interactions, sirtuin 1, a NAD^+^-dependent HDAC whose substrate specificity includes histone proteins, has been suggested to be activated by some dietary components (for example resveratrol, a type of natural phenol present in grape skins). Sirtuin 1 mediates some of the effects of dietary restriction that delay or reverse some of the physiological changes associated with aging through effects on DNA methylation [32].

Diets high in these methyl-donating nutrients can rapidly impact gene expression, especially during early development when the epigenome is first established, and can have long-lasting effects in adult life. Studies in animals have reported that diets poor in methyl-donating folate or choline before or just after birth cause permanent hypomethylation of parts of the genome. In adults, a methyl-deficient diet decreases DNA methylation, but the changes are reversible when methyl is added back into the diet. Further, depending on the dietary supplements received by a pregnant mouse, her offspring may have a different phenotype due to differential gene methylation. In an example of epigenome-modifying chemicals, bisphenol A (BPA) is widely used to manufacture numerous plastic products including containers. The pups of adult mice fed BPA were more likely to have an unhealthy phenotype (yellow/obese, prone to cancer and diabetes) compared to those born from mothers fed BPA with supplemented methyl-rich nutrients like folic acid and vitamin B12 (brown, thin and healthy). In this case, maternal nutrient supplementation counteracted the negative effects of chemical exposure, underscoring the importance of a good diet rich in fruit and vegetables and other high-quality foods. Methyl-donating nutrients act as co-substrates for methyl group transfers; the pool of available methyl donors is an important regulator of both DNA and histone methylation capacity [33] and their production is also dependent on BPA’s epigenetic effects. In this mouse model, altered DNA hypomethylation could be alleviated by folic acid as a dietary methyl donor.

Another striking example of the effects of early diets on epigenetics with consequences on the phenotype can be found in honey bees. The sterile worker bee differentiates from the fertile queen depending on the larval diet through epigenetic changes in DNA methylation patterns. Larvae designated to become queens are fed exclusively with royal jelly, which contains epigenetically active ingredients that silences a key gene which itself silences a group of queen genes [12]. Furthermore, DNA methylation changes occur during ageing, and it has become evident that early life nutrition can modulate DNA methylation and influence longevity, in particular by inducing long-term changes in DNA methylation and other marks that affect susceptibility to a range of ageing-associated diseases [11]; that is, a form of “cellular memory” (see [32] for a review). In this regard, the lifespan of a queen bee is up to twenty times that of a worker.

### 2.2. Nutritional Epigenetics and Cancer

Folic acid and vitamin B12 are two examples of epigenetically active ingredients that play important roles in DNA metabolism and the maintenance of DNA methylation patterns via chemical reaction of a methyl product. In one in vivo study, dietary folate intake was positively correlated with p16 tumor suppressor gene expression, a critical cancer-associated gene with frequent silencing DNA methylation of its promoter [12]. Altered p16 gene expression was observed in aged mouse colons, consistent with the known decrease in DNMT expression with aging.

Low folate intake has been associated with hypomethylation and an increased risk of colorectal and pancreatic cancers [9,34]. There is a growing body of epidemiological evidence that folate modulates anticarcinogenic properties through epigenetic changes, as folate deficiency reduces the potential for DNA methylation, and abnormal DNA methylation is associated with many types of cancer. Diets rich in fruits and vegetables containing natural anti-oxidants can protect against cancer. The potential epigenetic effects of several nutritional components in addition to folate, mostly derived from vegetables, have been examined in a number of studies, which have shown reductions in DNA hypermethylation of critical genes resulting in tumor suppression [32,35]. For example, green tea contains polyphenols, which are natural compounds widely distributed in plant foods and with many biological activities including inhibition of DNA methylation.

A variety of dietary factors are potential HDAC and HAT modulators. Some, such as sulforaphane, an isothiocyanate found in broccoli sprouts, or diallyl disulfide, an organosulfur compound in garlic, have been shown to act as HDAC inhibitors [9,35], a class of epigenetic therapeutic described further below. Such epigenetic drugs have been used to treat cancers in clinical trials due to their mode of action in restoring cancer cell differentiation and rendering tumors more sensitive to conventional therapies [36]. Several in vitro studies using these compounds have shown anti-carcinogenic effects associated with HDAC inhibition and histone acetylation [12,37,38]. Table 1 summarizes some dietary components considered to have protective effects against cancer through different epigenetic modifications.

As noted above, miRNAs can regulate DNA methylation and histone modifications, but promoter methylation or histone acetylation can also modulate miRNA expression as part of a complex network with feed-forward and feedback loops. Dysregulated miRNA expression is associated with the development or progression of human cancers through alterations in cell proliferation and apoptosis, but methyl- and folate-deficient diets can also result in aberrant miRNA expression to exert similar, pro-cancer effects [12]. Specifically, miR-222 has been considered a potential biomarker of nutritional status in humans and is implicated in obesity. Certain dietary components may protect against cancer through miRNA regulation, such as curcumin and retinoic acid [12], the former present in some plants and commonly used as a dietary supplement and food flavoring, and the latter present in any vitamin A-rich food.

Dietary patterns, not only individual nutrients, also influence behavior and phenotype in offspring. For example, Western diets tend to be high in saturated fats, red meats, and empty carbohydrates but low in fresh fruits and vegetables, whole grains, seafood, and poultry. This diet has been linked to many diseases including hypertension, heart disease, diabetes, and obesity and it has generally been linked to an increased risk of cancer [39,40]. In a specific example, nonalcoholic fatty liver disease (NAFLD) is a major public health concern in western societies. Nonalcoholic steatohepatitis (NASH), a form of NAFLD, is characterized by lipid accumulation in hepatocytes, inflammatory cell infiltrates, oxidative stress, and fibrosis and can lead to cirrhosis or hepatocellular carcinoma. The effects of a Western diet [41] or a high fat diet [42] on NAFLD development has been investigated in rodents. The risk of NAFLD may increase through an imbalance in fatty acids (FAs) in the Western diet. For instance, substituting linoleic acid with α-linolenic acid or long chain n-3 polyunsaturated fatty acids and decreased the n-6:n-3 FA ratio in high fat, high fructose (HFHF) diet-induced NASH [43]. The data from this study showed that decreasing the n-6:n-3 ratio by introducing healthy fats prevented HFHF-induced NASH by attenuating oxidative stress and inflammation and restoring the antioxidant state.

Epigenetics, such as DNA methylation, may be involved in the impact the western diet has on the human body. The effects of maternal Western diet on offspring physical activity, gene expression, and phenotype were assessed in [44]. Interestingly, differences in F1 female offspring but not in F2 male and female offspring were observed, suggesting that changes in the F1 generation were related to in utero somatic reprogramming. Epigenetic effects of specific FAs have been investigated in a number of studies. Eicosapentaenoic acid (EPA) and arachidonic acid (AA) are products of essential FA metabolism; both FAs are involved in inflammation resolution. EPA has been long regarded as a protective FA, particularly in the light of the favorable cardiometabolic effects of fish oil. One study [45] demonstrated a strong association between whole peripheral blood DNA methylation and EPA and AA in two distinct human cohorts—lactating infants and adult men—of different ages and developmental stages, thereby linking EPA and AA to DNA hypermethylation. Another study [46] reported that maternal dyslipidemia caused significant epigenetic changes in placentas and fetal livers and also increased fetal liver triacylglycerol accumulation. It has been shown from animal experiments that cardiovascular and metabolic diseases, particularly in males, may develop from alterations in DNA methylation. A recent study demonstrated how parental diet may affect their offspring’s epigenetic modifications and lead to the development of cardiovascular and metabolic diseases and impact central nervous system plasticity [47]. This study demonstrated that mice exposed to a high-fat, high-sugar diet (HFHSD) prior to and during pregnancy led to DNA modifications of their offspring’s compensatory renin-angiotensin system (RAS), a hormone system that regulates blood pressure, fluid retention, and vascular resistance. In adulthood, offspring from HFHSD-exposed dams exhibited several differences compared to control counterparts including but not limited to a lower level of angiotensin converting enzyme-2 (ACE2) gene expression in the brain stem, kidney, and cecum and higher ACE2 gene activity in the hypothalamus. These data suggest that perinatal exposure to HFHSD resulted in epigenetic modifications of the compensatory brain RAS, potentially affecting plasticity of neuronal networks leading to autonomic dysfunction in the male offspring.

In conclusion, following a western diet even before the chid’s birth may lead to physiological dysfunction via epigenetic changes. Further investigations, such as looking at potential transgenerational transmission in F2 male and female mice by crossing F1 males (offspring from HFHSD-exposed dams) with regular diet-fed females (and the opposite) would be of particular interest.

**Figure 1:**
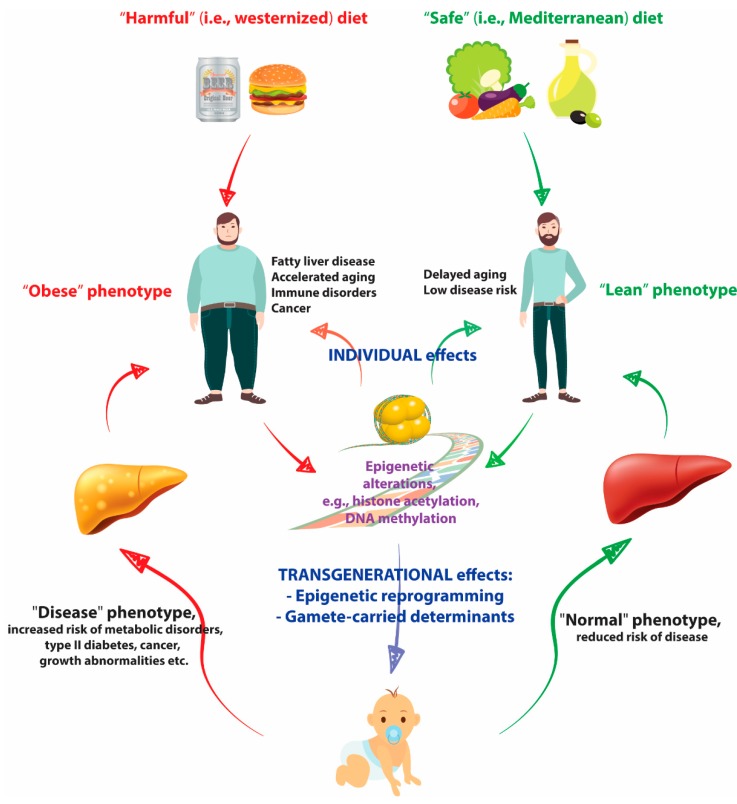
The diet affects the epigenome to modify individual and transgenerational phenotypes.

In contrast to the Western diet, a number of studies have described the health benefits of following the Mediterranean diet, which is associated with a reduced risk of heart disease and cardiovascular mortality as well as overall mortality. The Mediterranean diet traditionally includes fruits, vegetables, pasta and rice, fish and poultry, whole grains, and healthy fats (monounsaturated fats and polyunsaturated fats such as beneficial linolenic acid) and discourages red meat and saturated fats. The favorable effects of a Mediterranean diet as primary prevention of cardiovascular disease were assessed among persons at high cardiovascular risk [48]. The data from this study revealed that the incidence of major cardiovascular events was lower in those assigned to a Mediterranean diet supplemented with extra-virgin olive oil or nuts than among those assigned to a reduced-fat diet [48]. The Mediterranean diet is also associated with a reduced incidence of cancer, and the risk of breast cancer has been reported to be reduced in women who eat a Mediterranean diet supplemented with extra-virgin oil and mixed nuts. In relation to breast cancer, one study demonstrated that dietary patterns affect the mammary gland microbiome, establishing an alternative mechanistic pathway for breast cancer prevention [49]. The impact of maternal Mediterranean diet adherence during pregnancy on offspring behaviors has also been investigated, and maternal adherence to a Mediterranean diet in early pregnancy is associated with positive neurobehavioral outcomes in early childhood and with sex-dependent methylation differences in the control regions of imprinted genes [50]. Figure 1 summarizes how diet affects the epigenome to modify individual and transgenerational phenotypes.

### 2.3. Nutritional Epigenetics: The Future

Nutrients and bioactive food components can therefore reversibly alter the DNA methylation status, histone modifications, and chromatin remodeling, subsequently altering gene expression and having an impact on overall health. Bioactive food components, specific nutrients, and dietary patterns may have beneficial effects and overcome the negative impact of negative life behaviors, such as smoking or exposure to certain chemicals [51,52]. However, nutritional epigenetics is a quite recent subfield of epigenetics, so current knowledge on the precise effects of bioactive food components on epigenetics and their associations with phenotypes are limited. Deciphering the epigenetic signatures triggered by bioactive food components might pave the way for personalized nutritional interventions and aid our understanding of how our bodies respond to specific diets or nutrients [13]. For example, a recent study showed that fruit and juice epigenetic signatures as measured by DNA methylation marks are associated with independent immunoregulatory pathways, suggesting that the health benefits of fruit and juices are distinct. The identification of such differences between foods is the first step toward personalized nutrition [53].

## 3. Chemical Environmental Epigenetics

Environmental pollutants are among the environmental factors with epigenetic effects. Environmental exposures frequently considered epigenetic toxins include metals, such as arsenic, particulate matter from air pollution, organic compounds, pesticides, and other endocrine-disrupting chemicals, the latter reviewed in detail below.

### 3.1. Endocrine-Disrupting Chemicals (EDCs)

Endocrine-disrupting chemicals (EDCs) represent a group of environmental chemicals that interfere with hormone signaling pathways. Their actions on the epigenome are described as an exemplar. Humans and animals are regularly exposed to a wide range of EDCs. There is increasing evidence to suggest that EDC exposure during early life, in particular during fetal development, contributes to a variety of diseases, including cancers, which manifest later in life or even in the next generation. This suggests epigenetic inheritance of EDC-related events. Indeed, it is well established that epigenetic mechanisms play a central role in these long-lasting effects of EDCs. Moreover, a large number of studies have demonstrated that EDCs induce epigenetic changes. However, the underlying mechanisms of action are less well understood.

The effects of EDCs on epigenetics were recently reviewed in [33]. Briefly, the effects of EDCs can be global, that is, on epigenetic enzyme expression, i.e., DNMTs, HATs, and HDACs. In this case, for example, EDCs affect DNMT via nuclear hormone receptors such as the estrogen and androgen receptors or through miRNA expression, the latter being concomitant with other gene expression changes. The second mode of action of EDCs is gene-specific, regulating locus-specific epigenetic patterns. For example, EDCs may specifically affect one gene via nuclear receptors, which have been reported to regulate locus-specific chromatin states by recruiting histone modifiers and changing DNA methylation patterns by interacting with DNMTs and recruiting them to specific genomic sites. In addition, EDCs are likely to regulate other ncRNA expression via interference with nuclear receptors, which have a putative role in the regulation of ncRNA expression.

#### 3.1.1. EDC Exposure Induces Abnormal Development, Behavior, and Disease

The main consequences of environmental exposure to chemicals such as EDCs are adverse effects on the growth and development of reproductive organs, effects on neuronal differentiation, and an increase in the combined incidence of all cancers. EDCs are exogenous agents that alter one or more endocrine system axes and, consequently, impose adverse health effects at the level of the organism, its progeny, and/or subpopulations [54]. These compounds mimic natural hormones and are reported to promote a strong hormonal imbalance that causes developmental and reproductive disorders and tumor formation in children and adults after fetal exposure. An association between industrial chemicals/drugs and an elevated incidence of neoplasms in experimental animals and humans after early-stage exposure has also been described. According to the World Health Organization (WHO) [55], in addition to an elevated incidence of a variety of childhood and adult cancers, many different adult diseases have fetal origins but the causes have remained obscure. Reproductive disorders including testicular cancer, impaired semen quality, and the timing of puberty; altered brain and neurocognitive development; and adverse effects on adipose tissue, the adrenal glands, and the endocrine pancreas can be caused by prenatal EDC exposure. Sexual differentiation and reproductive functions are specifically under hormonal control (i.e., androgens and estrogens). Thyroid hormones are of special importance in brain development and are essential for regulating normal metabolism.

In terms of EDC-associated behavioral disorders, several studies have reported the BPA exposure affects behavior and development across several generations, including those not even exposed, depending on the window of exposure and exposure time. Sexual, social, learning, and maternal behavior changes are all associated with prenatal exposure to very low BPA doses, while exposure at different ages may also alter behavior. One study investigated the impact of low-dose BPA exposure on rat development and behavior over three generations, in which one group was not exposed to BPA and the other two were exposed at different periods of their life [56]. BPA exposure caused changes and defects over generations, including in unexposed offspring, implicating epigenetic changes. In particular, non-exposed offspring had body weight increases and changes in taste preferences compared to treated groups.

#### 3.1.2. EDCs, Epigenetics, and Cancer

Since the 1960s, data from numerous reproductive and developmental toxicity tests have provided growing evidence of the harmful impact of some environmental substances that interfere with normal endocrine function in humans.

In 2003, Birnbaum and Fenton [57] reviewed the consequences of exposure to endocrine disruptors on cancer and development and reported an association between prenatal exposure to diethylstilbestrol (DES), a synthetic estrogen originally used to prevent miscarriage, with a rare neoplasm, clear cell adenocarcinoma of the vagina. The data from animal studies of environmental estrogen exposure are particularly strong and correlate well with the human data. Earlier, McLachlan, et al. [58] demonstrated that prenatal DES exposure in mice resulted in uterine tumors and genital tract abnormalities in adulthood. Several other adverse effects of DES were subsequently found both in boys and girls [22,23], and Palmer, et al. [59] reported that gestational and perinatal exposure of female fetuses to DES was associated with an increased risk of breast cancer in the children of these exposed women. It was previously shown that elevated natural estrogen levels during gestation was associated with an increased incidence of breast cancer in the offspring later in life [60]. The naturally occurring phytoestrogen genistein found in most soy products has been shown to increase carcinogen-induced mammary cancers in female rat offspring after maternal genistein injection, suggesting that an elevated estrogenic environment in utero could enhance subsequent breast cancer risk at maturity [61]. The male offspring born to mothers treated with DES during pregnancy displayed feminization [62,63] and developmental malformations of the genital tract, infertility, and obesity [64]. There is also evidence of second-generation effects of DES on developmental abnormalities and tumorigenesis, as the sons of women exposed in utero had a higher prevalence of hypospadias than other case-controlled males [55]. Furthermore, experimental studies in rodents had already suggested that the carcinogenic effects of DES may be transmitted to succeeding generations, suggesting the involvement of epigenetic changes [65]. While the reproductive performance of the DES (F2) male mice was unaltered, an increased incidence of reproductive tract tumors was transmitted from the DES “grandmothers” to subsequent generations. DES (F2) female mice had an increased incidence of malignant reproductive tract tumors, including uterine adenocarcinomas; however, the fertility of these DES lineage female mice was not affected by DES exposure of their “grandmothers”, in contrast to the reduced fertility observed in their mothers (F1 DES-exposed females) [66]. The increased incidences of testicular and prostate cancers observed in European and American populations over the last 50 years may also be related to early exposure to environmental estrogens and/or anti-androgens [21,22,23,67]. Environmental anti-androgens are also associated with an increased incidence of gynecomastia in adult males, an effect similar to that observed after exposure to low dose DES during treatment of castration-resistant prostate cancer [68]. There is also growing evidence for an association between in utero exposure to various environmental toxins and childhood cancers, in particular brain tumors and hematological malignancies such as acute lymphoblastic leukemia (ALL), lymphoma, and leukemia [57].

Environmental dioxins, a type of EDC, have been called the most toxic synthetic compounds and can cause a host of adverse health effects. These pollutants mimic estrogen. As developmental and reproductive toxins, dioxins can cause immunotoxicity, dermal and hepatic toxicity, endocrine effects, and cancer [69]. Dioxin, the prototypical dioxin, is a human carcinogen [70,71] that increases the incidence of all cancers. Lung cancer, hematological cancers, and soft-tissue carcinomas have all been reported to develop after dioxin exposure. Humans are exposed in real life and on a daily basis to a mixture of structurally diverse EDCs that induce effects at different doses, at different ages, and on different target tissues.

Some studies have reported an increased risk of cancers in individuals exposed to high amounts of polycyclic aromatic hydrocarbons (PAHs), which are organic compounds produced when meats are grilled or barbecued [72,73] and considered a type of EDC. PAHs have been linked to many types of cancers in well-established animal models [74]. Some carcinogenic PAHs are genotoxic and induce mutations that initiate cancer; others are not genotoxic and instead affect cancer promotion or progression [75]. The pathways that mediate cell signaling for the endocrine disruptions induced by these chemicals include but is not limited to regulatory mechanisms (chromatin/epigenetic regulation) and cell function (development/differentiation, immune response/inflammation, apoptosis) [76]. Multiple epidemiological studies of people living in Europe, the United States, and China have linked in utero exposure to PAHs, through air pollution or parental occupational exposure, with poor fetal growth, reduced immune function, and poorer neurological development [77,78,79,80].

Therefore, there is evidence that EDCs can affect the epigenetic landscape of cells in target tissues by altering DNA methylation or histone methylation patterns, thereby affecting global gene expression profiles and transcriptomes. Given that epigenetic patterns can be heritable, these effects have been observed in successive generations [68].

### 3.2. Other Toxin Exposures and Epigenetic Effects

While smoking and alcohol consumption mainly cause disease through mutagenic events, both can also exert cellular effects through epigenetics. Children exposed to prenatal maternal smoking have epigenetic modifications in buccal mucosal cells. Maternal cigarette smoking during pregnancy has been investigated with respect to consequences on health, neurodevelopment, behavior, and cognitive function in infants. Maternal tobacco smoking during pregnancy is associated with respiratory diseases associated with immune system dysregulation such as asthma and allergies as well as cancer later in the life of offspring [26,81]. In another example, alcohol use is a risk factor for many cancers including oral, pharyngeal, laryngeal, esophageal, liver, colorectal, and breast cancer, and the risk of cancer is dose-dependent. Alcohol has deleterious/hazardous effects on growth, metabolism, and neuronal development through laying down epigenetic marks at early embryonic exposure, as evidenced by in vitro and animal studies. These disturbances in the epigenetic landscape may contribute to defects in certain memory and learning processes as well as malformations and abnormal fetal development. Numerous studies have reported that both genetic and environmental risk factors also play a role in the development of alcoholism [82,83].

## 4. Environmental Epigenetics and Cancer: Breast Cancer as a Specific Example

EDCs affect male mammary gland development [84] with transgenerational effects [85]. Alterations have been described in male and female mammary gland morphogenesis after BPA exposure that may contribute to breast cancer risk [86,87]. Fetal exposure to the estrogen-mimetic BPA has recently been shown to trigger epigenetic changes in postnatal and adult rat mammary glands throughout life and alter gene expression [88]. Indeed, changes in DNA methylation status or chromatin modifications in BPA-exposed animals may underpin some pre-neoplastic lesions, such as intraductal hyperplasias and neoplastic lesions. Animals exposed to BPA in utero have an increased risk of mammary carcinogenesis, which further increases when animals are exposed to a second carcinogenic stimulus. Dhimolea et al. (2014) showed that in utero BPA exposure altered both the DNA methylation profile and chromatin remodeling. In particular, increased levels of histone H3 lysine 4 trimethylation (H3K4me3), an established epigenetic marker of actively transcribed genes, was observed at the alpha-lactalbumin promoter in BPA-exposed animals. Global changes in mRNA expression profiles following fetal exposure to BPA were observed at sexual maturity, together with histological alterations of intraductal hyperplasia and ductal carcinoma in situ (DCIS). Interestingly, the BPA-treated group showed increased expression of c-Jun and downregulation of the cyclin-dependent kinase (CDK) inhibitor p57 (Cdkn1c), both known to be involved in cell cycle regulation. Kim et al. (2014) demonstrated that some environmental chemicals (e.g., methoxychlor (MXC), an estrogenic pesticide, and triclosan (TCS), a broad-spectrum antibacterial and antifungal agent widely used in hygiene products) stimulated ovarian cancer growth via upregulation of cyclin D1 and downregulation of p21 [89]. Prenatal exposure to the estrogen-mimetic dioxin delayed mammary gland proliferation and differentiation following puberty [90] and consequently increased sensitivity to carcinogens, because terminal end buds undergoing rapid proliferation and differentiation are more susceptible to tumor initiation [91]. Interestingly, dioxin exposure was associated with delayed breast development in girls [92]. Similar in utero effects were seen with atrazine (ATR), another environmental toxin and estrogen mimetic used as an herbicide and the most common member of the chlorotriazine family, the most heavily used agricultural pesticide. Following in utero exposure, ATR delayed early mammary gland development in female rat offspring, leading to increased terminal end buds and prolonging the window of sensitivity to potential carcinogens. A significant correlation between exposure to estrogens, phytoestrogens, and high fat diets and breast cancer susceptibility has been reported in several epidemiological studies. For instance, gestational/lactational exposure to genistein and vinclozolin, a phytoestrogen present in mammalian diets and an anti-androgenic agricultural fungicide, respectively, altered mammary gland structures of animals at puberty and in estrous. Epithelial proliferation was increased in EDC-exposed animals, as evidenced by increased Ki67 mRNA levels, epithelial bridging, ductal branching and hyperplasia, loss of epithelial cell polarization, and periductal fibrosis. Alterations in mammary gland structures were profound and particularly affected the terminal end buds, ducts, and lobuloalveolar structures. These modifications involved epigenomic changes and signatures that may influence subsequent breast cancer risk [93]. Indeed, a supplemental transcriptomic study showed alterations in genes related to development, differentiation, morphogenesis, and metabolism in the mammary glands of EDC-exposed rats compared to those in the mammary glands of controls, as well as accompanying histological modifications [94].

There are also reports of adult-onset transgenerational disease and histopathological changes (of the testis, prostate, and kidney) in F3 animals (eliminating the possibility of direct exposure effects) after exposure of a gestating F0 generation female to plastic-derived chemicals [95]. Vinclozolin is known to promote transgenerational epigenetic inheritance of adult-onset diseases in the rat by promoting male germline epigenome reprogramming [96,97,98]. Subsequently, all tissues propagated from these sperm have differentially damaged epigenomes and transcriptomes that can then alter phenotypes and influence the development of adult-onset diseases in subsequent generations [97].

## 5. The Potential of Epigenetic Drugs

Epigenetic regulation is highly complex. Histone proteins can be modified by several chemical modifications, while DNA is modified through the introduction of methyl groups to CpG islands. For each modification, two enzymatic families with competing activities add or remove chemical groups to affect chromatin structure and thus gene transcription and DNA repair. Interested readers are referred to an excellent review on the types of histones modifications, DNA methylation, and their regulating enzymes [99].

Abnormal epigenetic regulation may occur due to mutations in these enzymes or their altered activity as evidenced in a broad range of solid and hematological malignancies. These events participate in many diseases, including cancer. Therefore, the discovery of regulatory epigenetic enzymes has prompted the development of new therapeutic approaches over recent years, mostly in cancer research. Here we review drugs that acetylate histones and HDAC inhibitors, a primary class of epigenetic therapy showing good efficacy in cancer.

Histone acetylation is regulated by histone acetyltransferases (HAT) and HDAC, which add or removing acetyl groups from histone proteins to confer either an open or condensed chromatin conformation, respectively. Histone acetylation is generally associated with transcriptional activation, and many genes have been reported to be epigenetically silenced in cancer cells. In addition to histones, several non-histone proteins including many oncogenes and tumor suppressors such as Myc, p53, and PTEN are also dynamically acetylated. There are 11 Zn-dependent HDACs, which are subdivided into four classes: class I comprises HDACs 1, 2, 3, and 8; class IIA HDACs 4, 5, 7, and 9; class IIB HDACs 6 and 10; and class IV HDAC 11. Class I enzymes are expressed in the nucleus, class IIB in the cytoplasm, and class IIA shuttle between the cytoplasm and nucleus. HDAC inhibitors act by inhibiting enzymes responsible for transcriptional repression and silencing, thereby reversing the gene repression observed in malignancies. They might also help re-establish epigenetic control, such as in cancer patients with HAT gene mutations indicating decreased tumor acetylation.

These drugs can induce growth arrest, differentiation, and apoptosis in malignant cells. Vorinostat and romidepsin have been approved by the US Food and Drug Administration for the treatment of blood cancers, in particular myelodysplastic syndromes, leukemia, and lymphoma, and multiple HDAC inhibitors are currently in clinical trials for solid tumors [99], some in combination with either conventional therapies or newer, targeted therapies. However, these drugs have a more global genomic effect rather than targeting a single molecule or pathway. This continues to pose significant challenges in dissecting the specific effects of these drugs on histone and non-histone proteins. Nevertheless, the effects of epigenetic drugs seem to be quite well-tolerated. Furthermore, these epigenetic drugs have recently offered an exciting novel strategy to reverse immune suppression and restore T-cell-mediated anti-tumor activity. A summary of the underlying key mechanisms of epigenetic drugs that ‘prime’ tumors for immunotherapy is presented in [100]. In addition, these drugs may act as immunomodulatory agents in the treatment of cutaneous T cell lymphoma, in particular by decreasing the expression and secretion of the immunosuppressive cytokine IL-10 and by increasing the expression of the pro-inflammatory cytokine IFN-γ [101].

## 6. Conclusions

The health of an individual depends on the interaction of many environmental factors with its genetics. Lifestyle factors are likely to affect human health and offspring via epigenetic mechanisms. Variations in gene expression are influenced by epigenetics, which depends on our life experiences and habits including nutrition, behavior, and environmental toxin exposures. Chronic environmental exposures are thought to be partly responsible for the increased rates of all cancers, not only for those directly exposed, but also for the fetus. Some periods of development are particularly susceptible to the harmful effects of these toxins. In addition, the combination of toxin, dose, and critical exposure window must be considered but may be difficult to predict. Following toxin exposure, epigenomic alterations in the offspring may affect several organs and make the individual more sensitive to carcinogens during childhood or adulthood and increase disease susceptibility later in life, including cancer, diabetes, and autism. Epigenetic marks are influenced by the environment and these changes may be re-established in subsequent generation, or transgenerational epigenetic inheritance. Some of our traits, behaviors, illnesses, and good and bad experiences leave epigenetic tags that may be inherited; however, they may also be removed by lifestyle changes.

There is increasing evidence that different epigenetic mechanisms (DNA methylation, histone modifications, ncRNAs) are interconnected and form an “epigenetic network”. The observed phenotype—including global health and age-related processes—is the end result of a number of integrated signals. Lifestyle factors and environmental exposures leave epigenetic marks on our DNA that impact gene expression; some have protective effects while others are harmful. Fruit- and vegetable-rich diets may have similar effects on DNA to epigenetic drugs. A deeper understanding of epigenetic effects and the signaling pathways activated by bioactive food components would aid in assessing the role and potential benefit of nutrients on our health and reducing cancer susceptibility. Nutritional epigenetics could be combined with drugs for synergistic effects for treatment or prophylaxis or be adapted for pregnant woman to reduce the burden of chronic disease in offspring through an “epigenetically healthy” gestational diet. In both developed and developing countries, optimizing maternal diet is a challenging public health problem. Future work in the field of nutrition and epigenetics has the potential to provide significant benefit to public health, and personalized nutrition could become a part of a patient’s medical program.

The same applies to environmental chemicals. Due to their harmful effects on individuals, children, and possibly across generations, the impact of chemicals with epigenetic effects has become a major global public health concern that needs to be addressed. Some checkpoints or key molecules in the cell at the interface between the cell cycle and differentiation processes are likely to be targets of chemicals that favor cancer development. Understanding the epigenetic effects of EDCs would aid in assessing their action and mapping such marks could be used for risk management based on pathway-based toxicity testing.

## 7. Take-Home Messages

- Nutrition—in particular maternal diet and dietary patterns—and chemical pollutants are two important environmental factors that impact human health.
- These factors have a direct impact on the individual by contributing to the pathogenesis of many diseases, not least cancer.
- Furthermore, these factors probably span generations through epigenetic transmission, making them a major global public health problem not only for the individual but also future generations and society.
- Understanding the molecular mechanisms and signaling pathways involved in environmental epigenetics paves the way for both public health and targeted interventions to reduce their societal impact.

## Figures and Tables

**Table 1:** A summary of some dietary components considered to have protective effects against cancer.

Nutrient	Food Origin	Epigenetic Role
Methionine	Sesame seeds, brazil nuts, fish, peppers, spinach	SAM synthesis
Folic Acid	Leafy vegetables, sunflower seeds, baker’s yeast, liver	Methionine synthesis
Vitamin B12	Meat, liver, shellfish, milk	Methionine synthesis
Vitamin B6	Meats, whole grain products, vegetables, nuts	Methionine synthesis
SAM-e (SAM)	Popular dietary supplement pill; unstable in food	Enzymes transfer methyl groups from SAM directly to the DNA
Choline	Egg yolks, liver, soy, cooked beef, chicken, veal and turkey	Methyl donor to SAM
Betaine	Wheat, spinach, shellfish, and sugar beets	Break down the toxic byproducts of SAM synthesis
Resveratrol	Red wine	Removes acetyl groups from histones, improving health (shown in lab mice)
Genistein	Soy, soy products	Increased methylation, cancer prevention, unknown mechanism
Sulforaphane	Broccoli	Increased histone acetylation turning on anti-cancer genes
Butyrate	A compound produced in the intestine when dietary fiber is fermented	Increased histone acetylation turning on ‘protective’ genes, increased lifespan (shown in the lab in flies)
Diallyl sulphide (DADS)	Garlic	Increased histone acetylation turning on anti-cancer genes
